# Case reports of latent HBV hepatitis in patients after neurosurgical treatment for hypothalamic and pituitary tumors

**DOI:** 10.1186/s12879-020-04971-2

**Published:** 2020-03-18

**Authors:** Kuniyasu Niizuma, Yoshikazu Ogawa, Takayuki Kogure, Teiji Tominaga

**Affiliations:** 1 Department of Neurosurgical Engineering and Translational Neuroscience, Graduate School of Biomedical Engineering, Tohoku University, Sendai, Miyagi Japan; 2 Department of Neurosurgical Engineering and Translational Neuroscience, Tohoku University Graduate School of Medicine, Sendai, Miyagi Japan; 3 Department of Neurosurgery, Tohoku University Graduate School of Medicine, Sendai, Miyagi Japan; 4 Department of Neurosurgery, Kohnan Hospital, 4-20-1 Nagamachiminami, Taihaku-ku, Sendai, Miyagi 982-8523 Japan; 5 Department of Gastroenterology and Hepatology, Tohoku Medical and Pharmaceutical University, Sendai, Miyagi Japan

**Keywords:** Hepatitis B virus, Hypothalamic tumor, Pituitary tumor, Reactivation, Steroid

## Abstract

**Background:**

Hepatitis B virus (HBV) infection is a major public health problem worldwide. More than 2 billion people have been exposed to HBV, and about 257 million individuals are chronic carriers of HBV. HBV reactivation has been increasingly reported in HBV carriers who have undergone immunosuppression or chemotherapy, resulting in mortality. Treatment of hypothalamic/pituitary tumors in HBV carriers requires extensive care to avoid HBV reactivation as steroid therapy is required after surgery for hypothalamic/pituitary tumors.

**Case presentation:**

This retrospective review identified 5 patients, who were HBV carriers positive for hepatitis B surface antigen among 1352 patients with surgically treated hypothalamic/pituitary tumor in Kohnan Hospital between February 2007 and April 2017. Transsphenoidal surgery was performed with particular attention to prevent damage to the pituitary gland, with delicate manipulation to minimize postoperative steroid coverage. All patients received nucleot(s)ide analogue to control HBV-DNA levels before the surgery. As a result, all patients had a good clinical course. Blood examinations found a transient increase of liver enzymes and HBV-DNA levels in all patients, which started to decrease within 2 weeks after surgery. No specific treatment other than nucleot(s)ide analogues was needed to maintain liver function, and all patients returned to their previous activities including reinstatement.

**Conclusion:**

Initiation of nucleot(s)ide analogues administration prior to the surgery for hypothalamic/pituitary tumors can be an effective strategy for preventing reactivation in HBV carriers. Appropriate screening of the patient’s HBV phase, optimal timing of nucleot(s)ide analogues -administration, and administration period of nucleot(s)ide analogues need to be established.

## Background

Hepatitis B virus (HBV) infection is a major public health problem worldwide. More than 2 billion people have been exposed to HBV, and about 257 million individuals are chronic carriers of HBV [1–3]. Around 10% of infected people develop chronic infection [2, 4, 5], but most remain asymptomatic. However, 20–30% of individuals with chronic infection develop chronic liver diseases such as cirrhosis and/or liver cancer [3]. HBV caused 887,000 deaths in 2015, mostly of complications including cirrhosis and hepatocellular carcinoma. Combined with other types, viral hepatitis caused the deaths of 1.34 million people, comparable with the number of deaths caused by tuberculosis [6]. The most common route of transmission is perinatal or horizontal [7], and universal vaccination is recommended [3].

HBV reactivation has been increasingly reported in patients with HBV in phases of inactive carrier and resolved hepatitis B (HBs-Ag negative and HBs-Ab positive), who have undergone immunosuppression including steroids [8–10] or chemotherapy [11–14]. Reactivation associated with immunosuppressive therapy tends to progress to severe acute exacerbation, and leads to hepatic failure and death [15–17]. HBV reactivation greatly hinders the treatment of underlying diseases, so the establishment of a preventive strategy for HBV reactivation in high-risk patients is extremely important.

Not only the administration of drugs to affect the immune status of the host, but the alteration of endogenous steroid secretion such as Cushing’s syndrome can also be a cause of HBV reactivation [18]. Surgeries for hypothalamic/pituitary tumors could be a great risk for reactivation due to perioperative administration and a change of endogenous steroid secretion.

This study retrospectively analyzed asymptomatic carriers of HBV who underwent removal of hypothalamic/pituitary tumor with postoperative steroid therapy. Perioperative management to avoid HBV reactivation in collaboration with hepatologists is discussed.

## Case presentation

We retrospectively reviewed 1352 patients with hypothalamic/pituitary tumors who underwent surgical removal in Kohnan Hospital between February 2007 and April 2017. Six patients (4 men and 2 women) were preoperatively diagnosed as HBV carriers based on a positive reaction for hepatitis B surface antigen (HBsAg). One male patient was lost to follow up and was excluded. The detailed medical records of the remaining 5 patients, including perioperative management and postoperative courses, were extracted from our clinical database and analyzed. The mean age was 59.4 ± 8.0 years (range, 47–69 years). All tumors were located in the sella up to the suprasellar cistern, and manifested as a visual disturbance. One patient with craniopharyngioma had a disturbance of consciousness due to occlusive hydrocephalus. Before the referral to our hospital, one patient with Cushing’s disease had already started the administration of nucleot(s)ide analogue (NA) to treat HBV reactivation in inactive carrier (Tables 1, 2). The cause for the reactivation seemed to be an excessive endogenous secretion of steroids due to Cushing’s disease. Other four patients’ phase of HBV was inactive carrier and had started the administration of NAs after the referral to our hospital through the transsphenoidal surgery. Before the surgery, the HBV- deoxyribonucleic acid (DNA) level was decreased to below the lower detection sensitivity in all patients except in one patient (case 1) that lacked the result of HBV-DNA just before the surgery. The median duration of NA administration before the surgery was 22 days (range 14–224 days) (Table 2). According to the clinical parameter and the imaging such as abdominal ultrasound, all five patients had no sign to indicate the existence of advanced fibrosis in the liver.

**Table 1:** Clinical characteristics of patients

No.	Sex	Age (year)	Tumor pathology	Phase of HBV infection	HBeAg / HBeAb	HBs-antigen (qualitative or IU/ml)
1	F	62	gonadotroph cell adenoma	inactive carrier	- / +	+
2	F	47	corticotroph cell adenoma	reactivation	- / +	> 250
3	M	69	plurihormonal cell adenoma	inactive carrier	- / +	> 250
4	M	59	gonadotroph cell adenoma	inactive carrier	- / +	+
5	M	60	craniopharyngioma	inactive carrier	- / +	202

*HBV* hepatitis B virus, *M* male, *F* female

**Table 2:** ALT and HBV-DNA level before and after transsphenoidal surgery with prophylactic NA administration

No.	HBV-DNA level before NA administration (log IU/ml)	ALT before NA (IU/l)	HBV-DNA level before surgery (log IU/ml)	ALT before surgery (IU/l)	Duration of steroid administration after surgery	Types of NA	Duration of NA administration before surgery	Duration of NA administration after surgery
1	2.1	27	not available	23	19 days	lamivudine	14 days	6 weeks
2	> 8.3	911	not detected	194	3 days	entecavir	22 days	2 years
3	2.6	24	not detected	19	2 days	entecavir	224 days	continued
4	not detected	18	not detected	16	continued	entecavir	14 days	continued
5	2.3	57	not detected	48	continued	tenofovir	23 days	continued

*HBV* hepatitis B virus, *DNA* deoxyribonucleic acid, *NA* nucleot(s)ide analogues, *ALT* alanine aminotransferase

Administration of NA was skipped only one time (the evening on the day of surgery). To minimize hypopituitarism, the tumor was carefully dissected from the pituitary gland, which enabled us to decrease the dose and duration of the postoperative steroid coverage.

After the surgery, the HBV-DNA levels transiently increased with elevation of alanine aminotransferase (ALT) in four patients (case 2–5) however no specific treatment other than NAs was needed to maintain liver enzymes (Table 1). The administration of NA was continued after the surgery for two patients. One of the patients (case 4) developed postoperative adrenal insufficiency and needed to administer low dose steroid continuously. The other patient (case 5) with a craniopharyngioma originated from pituitary stalk also needed to continue low dose steroid administration. For other three patients, steroid administration was discontinued early after the surgery (2 days, 3 days and 19 days) and the NA administration was stopped after the HBV-DNA level decreased to below the lower detection sensitivity for 2 patients (case 1 and 2) and the cessation of the administration is planned for one patient (case 3). All patients returned to their previous activities including reinstatement, and the patients were followed up by both neurosurgical and hepatological services.

**Fig. 1:**
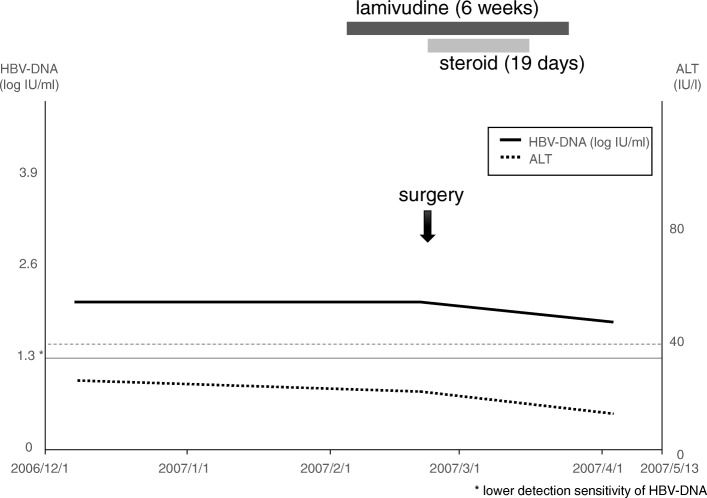
Clinical course of case 1, 62-year-old female, gonadotroph cell adenoma. Lamivudine was administered 2 weeks before transsphenoidal surgery and discontinued 6 weeks after the surgery. Steroid coverage was discontinued 19 days after the surgery

**Fig. 2:**
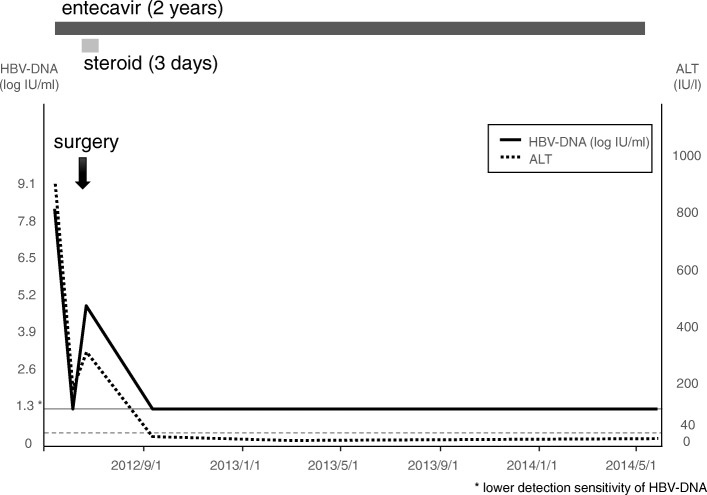
Clinical course of case 2, 47-year-old female, corticotroph cell adenoma. Entecavir was administered 22 days before transsphenoidal surgery and discontinued 2 years after the surgery. HBV-DNA level was temporary increased after the surgery, and immediately decreased to lower detection sensitivity. Steroid coverage was discontinued 3 days after the surgery

**Fig. 3:**
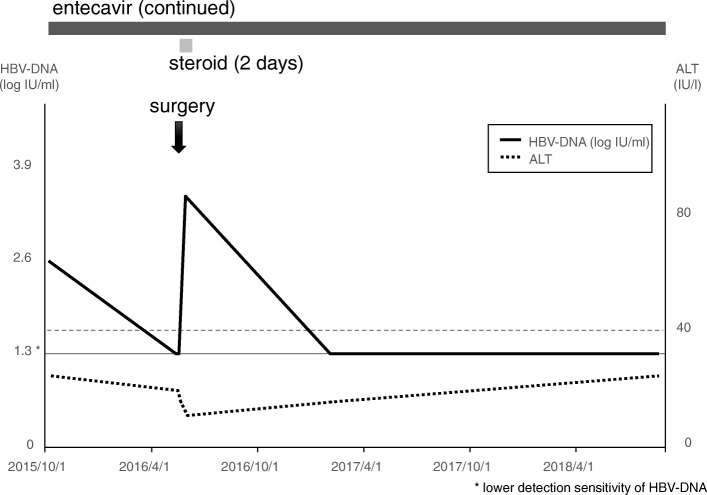
Clinical course of case 3, 69-year-old male, plurihormonal cell adenoma. Entecavir was administered 224 days before transsphenoidal surgery and transsphenoidal surgery was performed. HBV-DNA level was temporary increased after the surgery, then decreased to lower detection sensitivity. Cessation of the administration is planned. Steroid coverage was discontinued 2 days after the surgery

**Fig. 4:**
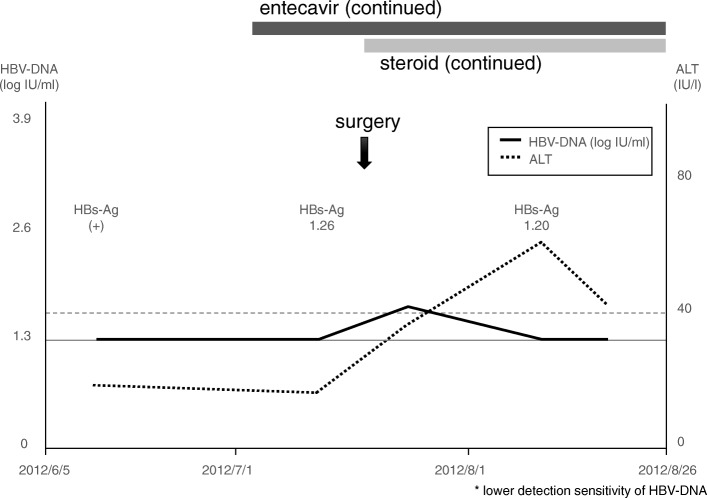
Clinical course of case 4, 59-year-old male, gonadotroph cell adenoma Entecavir was administered 2 weeks before transsphenoidal surgery. Low dose steroid coverage and entecavir were continued because of the continued adrenal failure.

**Fig. 5:**
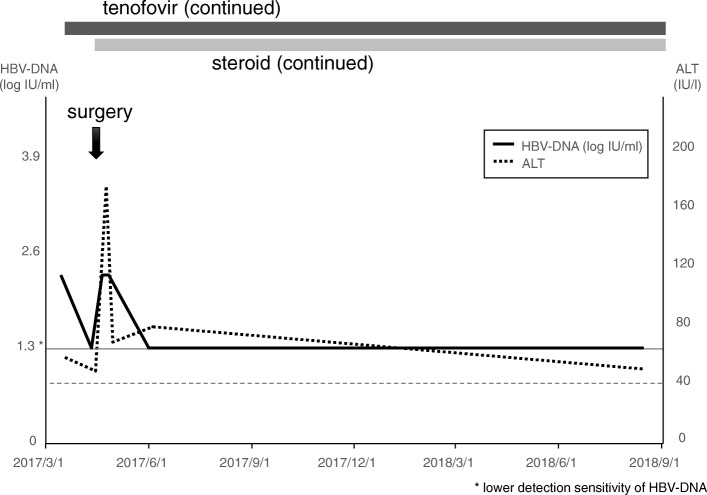
Clinical course of case 5, 60-year-old male, craniopharyngioma. Tenofovir disoproxil fumarate was administered 23 days before extended transsphenoidal surgery. A long-term coverage with low dose steroid was required after the surgery, thus tenofovir was also continued

Here we present the clinical course of each patient (Figs. 1, 2, 3, 4 and 5).

### Case 1 (Fig. 1)

A 62-year-old female, inactive HBV carrier, suffered from cardiogenic embolism. The administration of tissue plasminogen activator improved her symptoms. Brain magnetic resonance imaging (MRI) demonstrated sellar tumor, and she was referred to our service. Lamivudine was administered 2 weeks before transsphenoidal surgery and discontinued 6 weeks after the surgery. Steroid coverage was discontinued 19 days after the surgery.

### Case 2 (Fig. 2)

A 47-year-old female with HBV reactivation. She was diagnosed with HBV in inactive carrier phase at the age of 19. When she developed HBV reactivation, she was diagnosed with Cushing’s disease with sellar tumor by brain MRI, and excessive endogenous secretion of steroids seemed to be the cause of reactivation. Administration of entecavir was started and she was referred to our service. Transsphenoidal surgery was performed, and steroid coverage was discontinued 3 days after the surgery. HBV-DNA level was temporary increased after the surgery, and immediately decreased to lower detection sensitivity. Entecavir was discontinued 2 years after the surgery.

### Case 3 (Fig. 3)

A 69-year-old male who were treated with entecavir for HBV hepatitis. He suffered from visual disturbance. Brain and ophthalmological tests revealed sellar tumor and he was referred to our service. Transsphenoidal surgery was performed and steroid coverage was discontinued 2 days after the surgery. HBV-DNA level was temporary increased after the surgery, but decreased to lower detection sensitivity for 2 years, and cessation of the administration is planned.

### Case 4 (Fig. 4)

A 59-year-old male, inactive HBV carrier, with a history of partial resection of the sellar tumor 11 years ago. He suffered from general fatigue due to the parasellar tumor and adrenal insufficiency. He was referred to our service. He was considered as an inactive HBV carrier because HBV surface antigen was positive although the level of HBV-DNA was below the detection sensitivity. Transsphenoidal surgery was performed. Entecavir was started 2 weeks before transsphenoidal surgery. Low dose steroid coverage and entecavir were continued because of the continued adrenal failure.

### Case 5 (Fig. 5)

A 60-year-old male, inactive HBV carrier, suffered from gait disturbance and urinary incontinence. Brain MRI revealed suprasellar tumor and hydrocephalus, and he was referred to our service. Tenofovir disoproxil fumarate was started 3 weeks before the surgery. Extended transsphenoidal surgery was performed, and craniopharyngioma originated from pituitary stalk was totally resected. Ventriculoperitoneal shunt was also performed to resolve hydrocephalus. A long-term coverage with low dose steroid was required after the surgery, thus tenofovir was also continued.

## Discussion and conclusion

Both endogenous production and exogenous administration of corticosteroids should be controlled in HBV carriers. Spontaneous reactivation of HBV can occur in inactive HBV carriers even without steroid administration [19], and the risk factors reported were aging [19], diabetes mellitus [20], pregnancy [21], and emotional [22] or physical stress including surgeries [19]. Even the patient with steroid-producing tumors can cause HBV reactivation. HBV reactivation is reported in a patient with steroid-producing adrenal tumor followed by adrenalectomy that resulted in rapid improvement of HBV-DNA level and liver function [23]. HBV reactivation is also reported in a patient with Cushing’s syndrome. The patients could not receive adrenalectomy due to coagulopathy and poor status, which resulted in the death of hepatic failure and sepsis [15]. In our series, HBV reactivation was found in a patient with Cushing’s disease, which was successfully treated by the combination of antiviral therapy, transsphenoidal surgery, and gamma knife radiosurgery [22] (Case 2). Steroid-free chemotherapy decreases the incidence and severity of HBV reactivation in HBsAg-positive lymphoma patients [8].

Treatment of the hypothalamic/pituitary tumor of the HBV carriers must resolve the contradiction between steroid supplementation and the risk of HBV reactivation due to steroid therapy. Hypopituitarism is one of the serious complications after transsphenoidal surgery and/or gamma knife radiosurgery [24, 25], and steroid supplementation for some duration is essential. Neurosurgeons should be extremely careful not to damage the pituitary gland, with delicate manipulation in surgery, and ensuring adequate distance from the residual tumor to allow for subsequent gamma knife therapy [26]. However, even a very low dose steroid treatment can cause HBV reactivation in an inactive carrier. Reactivation is reported in an inactive HBV carrier with rheumatoid arthritis treated with 2.5 mg prednisolone/day that resulted in death [15]. Therefore, the minimization of the dose and duration of steroid administration may not be enough to avoid HBV reactivation. No standard procedure has been reported for the prevention of HBV reactivation in patients with hypothalamic/pituitary tumors receiving surgical resection. The American Association for the Study of Liver Diseases has a guideline for HBV [27] and HBsAg-positive patients should receive NAs as prophylaxis before the immunosuppressive or cytotoxic therapy, however, the guideline is intended for use in the long-term administration of drugs with a risk of reactivation. In our cases, HBV-DNA level increased after the surgery in 4 patients, however, no patients developed HBV reactivation. Two patients could stop administrating the NA safely. For patients in inactive HBV carrier, starting NAs prior to the surgery for hypothalamic/pituitary tumor would be suitable prophylaxis for HBV reactivation. Neurosurgeons should make their best efforts to manage NAs in collaboration with hepatologists.

Prophylaxis can be useful for the prevention of HBV reactivation in carriers [28, 29]. Prophylactic lamivudine reduced the incidence and severity of hepatitis and decreased hepatitis-related deaths during chemotherapy for non-Hodgkin’s lymphoma [28]. However, compared with ‘universal prophylaxis,’ ‘strict monitoring’ was more appropriate and cost-effective for HBV carriers with non-Hodgkin lymphoma during chemotherapy [30]. Serum HBV-DNA is the most sensitive indicator to predict HBV reactivation, so it should be monitored for a longer period, even after completion of initial treatments. In a series of 8 patients with de novo HBV-related hepatitis after chemotherapy, HBsAg seroreversion occurred at a median of 10 weeks after a 100-fold increase in serum HBV-DNA had occurred, followed by alanine aminotransferase deterioration [31].

The strategy to prevent HBV reactivation is not well established as described above, so indications for the patients, choice of NAs and duration of antiviral therapy have to be established. Further investigations are needed to determine the appropriate screening methods and preventive supplementation of NAs for HBV reactivation in patients with hypothalamic/pituitary tumors. Our results imply the effectiveness of preventive supplementation of NAs, small number of cases and lack of controls are major limitations.

In conclusion, the initiation of NAs administration prior to the surgery for hypothalamic/pituitary tumors can be an effective strategy for preventing reactivation in HBV carriers. Appropriate screening of the patient’s HBV phase, optimal timing of NAs-administration, and administration period of NAs need to be established.

## Data Availability

Because this manuscript is a case report there is no datasets, which could be freely available to use supporting the conclusions of this article.

## Abbreviations

HBV: Hepatitis B virus
NA: Nucleot(s)ide analogue
DNA: Deoxyribonucleic acid
ALT: Alanine aminotransferase
MRI: Magnetic resonance imaging

## References

[1] Lavanchy D (2010). Hepatitis B virus epidemiology, disease burden, treatment, and current and emerging prevention and control measures. J Viral Hepat.

[2] McMahon BJ (2010). Natural history of chronic hepatitis B. Clin Liver Dis.

[3] WHO (2017). Hepatitis B fact sheet.

[4] Fattovich G, Bortolotti F, Donato F (2008). Natural history of chronic hepatitis B: special emphasis on disease progression and prognostic factors. J Hepatol.

[5] Ganem D, Prince AM (2004). Hepatitis B virus infection--natural history and clinical consequences. N Engl J Med.

[6] WHO (2017). Global hepatitis report, 2017.

[7] Lok AS (2002). Chronic hepatitis B. N Engl J Med.

[8] Cheng AL, Hsiung CA, Su IJ, Chen PJ, Chang MC, Tsao CJ (2003). Steroid-free chemotherapy decreases risk of hepatitis B virus (HBV) reactivation in HBV-carriers with lymphoma. Hepatology..

[9] Lam KC, Lai CL, Trepo C, Wu PC (1981). Deleterious effect of prednisolone in HBsAg-positive chronic active hepatitis. N Engl J Med.

[10] Perrillo RP (2001). Acute flares in chronic hepatitis B: the natural and unnatural history of an immunologically mediated liver disease. Gastroenterology..

[11] Gupta S, Govindarajan S, Fong TL, Redeker AG (1990). Spontaneous reactivation in chronic hepatitis B: patterns and natural history. J Clin Gastroenterol.

[12] Lok AS, McMahon BJ (2009). Chronic hepatitis B: update 2009. Hepatology..

[13] Reddy KR, Beavers KL, Hammond SP, Lim JK, Falck-Ytter YT (2015). American Gastroenterological Association Institute guideline on the prevention and treatment of hepatitis B virus reactivation during immunosuppressive drug therapy. Gastroenterology..

[14] Serper M, Forde KA, Kaplan DE (2018). Rare clinically significant hepatic events and hepatitis B reactivation occur more frequently following rather than during direct-acting antiviral therapy for chronic hepatitis C: data from a national US cohort. J Viral Hepat.

[15] Bae JH, Sohn JH, Lee HS, Park HS, Hyun YS, Kim TY (2012). A fatal case of hepatitis B virus (HBV) reactivation during long-term, very-low-dose steroid treatment in an inactive HBV carrier. Clin Mol Hepatol.

[16] Chan HL, Tsang SW, Hui Y, Leung NW, Chan FK, Sung JJ (2002). The role of lamivudine and predictors of mortality in severe flare-up of chronic hepatitis B with jaundice. J Viral Hepat.

[17] Mori N, Suzuki F, Kawamura Y, Sezaki H, Hosaka T, Akuta N (2012). Determinants of the clinical outcome of patients with severe acute exacerbation of chronic hepatitis B virus infection. J Gastroenterol.

[18] Tsou PL, Lee HS, Jeng YM, Huang TS (2002). Submassive liver necrosis in a hepatitis B carrier with Cushing’s syndrome. J Formos Med Assoc.

[19] Kamitsukasa H, Iri M, Tanaka A, Nagashima S, Takahashi M, Nishizawa T (2015). Spontaneous reactivation of hepatitis B virus (HBV) infection in patients with resolved or occult HBV infection. J Med Virol.

[20] Chen CY, Tien FM, Cheng A, Huang SY, Chou WC, Yao M (2018). Hepatitis B reactivation among 1962 patients with hematological malignancy in Taiwan. BMC Gastroenterol.

[21] Singhal A, Kanagala R, Jalil S, Wright HI, Kohli V (2011). Chronic HBV with pregnancy: reactivation flare causing fulminant hepatic failure. Ann Hepatol.

[22] Tashiro R, Ogawa Y, Tominaga T (2017). Rapid deterioration of latent HBV hepatitis during Cushing disease and posttraumatic stress disorder after earthquake. J Neurol Surg A Cent Eur Neurosurg.

[23] Smith P. J., Suri D. (2011). Adrenalectomy to treat reactivated chronic hepatitis B infection in a patient with a steroid-secreting adrenal tumour. Case Reports.

[24] Fatemi N, Dusick JR, Mattozo C, McArthur DL, Cohan P, Boscardin J (2008). Pituitary hormonal loss and recovery after transsphenoidal adenoma removal. Neurosurgery..

[25] Gansauge F, Poch B, Kleef R, Schwarz M (2013). Effectivity of long antigen exposition dendritic cell therapy (LANEXDC®) in the palliative treatment of pancreatic cancer. Curr Med Chem.

[26] Ogawa Y, Tominaga T (2016). Risk of reactivation of latent HBV hepatitis in patients under neurosurgical treatment. J Neuroinfect Dis.

[27] Terrault NA, Lok ASF, McMahon BJ, Chang KM, Hwang JP, Jonas MM (2018). Update on prevention, diagnosis, and treatment of chronic hepatitis B: AASLD 2018 hepatitis B guidance. Hepatology..

[28] Hsu C, Hsiung CA, Su IJ, Hwang WS, Wang MC, Lin SF (2008). A revisit of prophylactic lamivudine for chemotherapy-associated hepatitis B reactivation in non-Hodgkin's lymphoma: a randomized trial. Hepatology..

[29] Saito S, Mukohara K (2008). Clinical needs of patients attending a women's health center in Japan. Intern Med.

[30] Masarone M, De Renzo A, La Mura V, Sasso FC, Romano M, Signoriello G (2014). Management of the HBV reactivation in isolated HBcAb positive patients affected with non Hodgkin lymphoma. BMC Gastroenterol.

[31] Hui CK, Cheung WW, Zhang HY, Au WY, Yueng YH, Leung AY (2006). Kinetics and risk of de novo hepatitis B infection in HBsAg-negative patients undergoing cytotoxic chemotherapy. Gastroenterology..

